# Theoretical Modeling of Cognitive Dysfunction in Schizophrenia by Means of Errors and Corresponding Brain Networks

**DOI:** 10.3389/fpsyg.2018.01027

**Published:** 2018-07-03

**Authors:** Yuliya Zaytseva, Iveta Fajnerová, Boris Dvořáček, Eva Bourama, Ilektra Stamou, Kateřina Šulcová, Jiří Motýl, Jiří Horáček, Mabel Rodriguez, Filip Španiel

**Affiliations:** ^1^National Institute of Mental Health, Klecany, Czechia; ^2^3rd Faculty of Medicine, Charles University in Prague, Prague, Czechia

**Keywords:** cognitive deficits, schizophrenia, cognitive tests, errors, brain networks, lesions, fMRI

## Abstract

The current evidence of cognitive disturbances and brain alterations in schizophrenia does not provide the plausible explanation of the underlying mechanisms. Neuropsychological studies outlined the cognitive profile of patients with schizophrenia, that embodied the substantial disturbances in perceptual and motor processes, spatial functions, verbal and non-verbal memory, processing speed and executive functioning. Standardized scoring in the majority of the neurocognitive tests renders the index scores or the achievement indicating the severity of the cognitive impairment rather than the actual performance by means of errors. At the same time, the quantitative evaluation may lead to the situation when two patients with the same index score of the particular cognitive test, demonstrate qualitatively different performances. This may support the view why test paradigms that habitually incorporate different cognitive variables associate weakly, reflecting an ambiguity in the interpretation of noted cognitive constructs. With minor exceptions, cognitive functions are not attributed to the localized activity but eventuate from the coordinated activity in the generally dispersed brain networks. Functional neuroimaging has progressively explored the connectivity in the brain networks in the absence of the specific task and during the task processing. The spatio-temporal fluctuations of the activity of the brain areas detected in the resting state and being highly reproducible in numerous studies, resemble the activation and communication patterns during the task performance. Relatedly, the activation in the specific brain regions oftentimes is attributed to a number of cognitive processes. Given the complex organization of the cognitive functions, it becomes crucial to designate the roles of the brain networks in relation to the specific cognitive functions. One possible approach is to identify the commonalities of the deficits across the number of cognitive tests or, common errors in the various tests and identify their common “denominators” in the brain networks. The qualitative characterization of cognitive performance might be beneficial in addressing diffuse cognitive alterations presumably caused by the dysconnectivity of the distributed brain networks. Therefore, in the review, we use this approach in the description of standardized tests in the scope of potential errors in patients with schizophrenia with a subsequent reference to the brain networks.

## Introduction

Cognitive dysfunction in schizophrenia has been propagated as a core component of the illness since Kraepelin (Kraepelin, 1919). Until now, there is no evident comprehension of the mechanisms of cognitive disturbances in schizophrenia. On the one hand, regional brain volume alterations in patients with schizophrenia as compared to healthy individuals, are associated with IQ-dependent cognitive measures, e.i verbal and non-verbal memory, processing speed (for review see Antonova et al., 2005). Though, the patterns of cognitive deficits seem to be more complex that the pattern of structural alterations. On the other hand, the dysconnectivity theory (referred to as “cognitive dysmetria”) of schizophrenia suggests that the cognitive deficits might originate from the aberrant functional brain networks activity (Andreasen et al., 1996). This disrupted connectivity results in altered functional integration since it involves either exaggerated connections or weakened pathways (Stephan et al., 2006; Fornito et al., 2011). The deficits in attention and working memory, as it was shown by Whitfield-Gabrieli et al, correlate with the alterations in networks coupling. Specifically, the lack of suppression of the default-mode network (DMN), which intends to suppress during information processing, implies the disbalanced excitatory/inhibitory brain circuits in schizophrenia (Whitfield-Gabrieli et al., 2009).

For the successful information processing, well-coordinated functioning of the distinct brain structures is essential (Pöppel, 1989). Neurological, neuropsychological and neuroimaging studies show that genuinely all cognitive functions rely on the enactments of the dispersed cortical and subcortical brain structures and are not restrained to the specific structures (Singer, 2013; Sporns, 2013). Presumably, cognitive operations arise out of a composed action in the brain networks. Given the multidimensional organization of cognitive functions, the identification of the neural networks responsible for the specific cognitive functions seems to be problematic, especially since the brain structures are oftentimes associated with a number of cognitive operations. The one-to-one relationship of specific brain region and cognitive function does not seem to hold true to the psychiatric diseases in general and to schizophrenia in particular.

### Definition of the qualitative characteristics of cognitive functioning in patients with schizophrenia

Neuropsychological studies of schizophrenia have a long history. The functional alterations are pronounced in motor and perceptual processes, spatial functions, verbal and non-verbal memory, attention and executive functioning (Green and Harvey, 2014). Consistent results across the studies demonstrated that if not all, the majority of schizophrenia patients perform more poorly than healthy controls (Mesholam-Gately et al., 2009). In accordance with Harvey (Harvey, 1997), the depth of the deficit can be described relatively to the corresponding reduction of performance in the number of SDs compared to the population norm and the number of affected functions as following: “mild,” 0.5–1.0 SD, perceptual capacity, remote memory; “moderate,” 1.0–2.0, remote, short and working memory, attention, and visuomotor functions; and “severe cognitive disability,” 2.0–5.0 SD, learning, executive function, memory, vigilance, motor functions, verbal fluency). Subgroups of individuals with SZ may cluster together according to their pattern of cognitive deficits, suggesting the existence of subtypes of dysfunction (Rodriguez et al., 2015). Previous findings agree on two extreme clusters, characterized by near-normal performance on one side (e.g., Goldstein et al., 1998) and profound global dysfunction on the other side (e.g., Goldstein and Shemansky, 1995). One or two remaining subsets are in agreement with the partial deficit (e.g., visual memory and processing speed in Gilbert et al., 2014) with mild cognitive impairment. Standardized scoring in the majority of the neurocognitive tests renders the index scores or the achievement indicating the severity of the cognitive impairment rather than the actual performance. Though such approach facilitates the tracking of the cognitive functioning during the follow-up, at the same time it may lead to the situation when two patients with the same index score of the particular cognitive test demonstrate qualitatively different performances. This may support the view that test paradigms that habitually incorporate different cognitive variables associate weakly, reflecting an ambiguity in the interpretation of noted cognitive constructs (Poldrack, 2011).

Therefore, qualitative approach, being focused on the performances, allows to characterize the types of errors and track of them throughout the testing procedure (Zaytseva et al., 2015). The commonality of the errors across the number of tests may identify the impaired brain structures or networks. Qualitative analysis of tests with the definition of errors (Golden et al., 2000; Strauss et al., 2006) is widely applied in patients with mild cognitive impairment or neurodegenerative diseases (for instance, see Collie and Maruff, 2002; Thompson et al., 2005) that are also known to present with generalized cognitive deficits. The qualitative characterization of cognitive performance might be beneficial in addressing diffuse cognitive alterations presumably caused by the dysconnectivity of the distributed brain networks. The present selective review is focused on the description of the errors in widely used cognitive tests and highlighting the specificities of the performance in schizophrenia patients, hence providing a reference frame for the search of the underlying neural mechanisms.

## Trail making test (TMT)^1^

### Behavioral performance (errors) and brain correlates of TMT in healthy and lesion cohorts

The trail making task includes two variants: TMT-A examines mainly visuoperceptual abilities and processing speed, TMT-B reflects working memory as well as task-switching ability. Two parameters, the time required to complete the test and the number of errors are typically used to measure performance in TMT (Lezak et al., 2012). Subtraction B-A or ratio B/A of the completion time are used to minimize visuoperceptual and working memory demands, thus specifically evaluating mental flexibility and executive control (Sánchez-Cubillo et al., 2009). A study in healthy elderly subjects (Oosterman et al., 2010) supported this functional construction of the TMT scores showing that the predictive value of individual neuropsychological test scores (working memory, executive function, speed and attention, episodic memory) differed among the various TMT-B variables. While the TMT-B total completion time was associated with all neuropsychological scores, only executive function predicted the ratio score (TMT-B/A). In terms of qualitative analysis, the following general categories of observed errors in TMT are omission, perseveration, repetition, sequential and proximity errors (Lezak et al., 2012). **Omission errors** apply mostly to TMT-A and refer to skipping a number in the sequence. **Perseveration errors**, referred to as set-shifting errors, are only seen in TMT-B, when the subject fails to change a set from number to letter and vice versa. **Repetition errors** are made when the same circle is selected more than once. **Sequential errors** can be seen in both TMT variants when the number or letter sequence is incorrect. **Spatial or proximity errors**, also known as capture responses, represent errors in the sequence which occur when the subject circles an incorrect number or letter that is located nearby. Most TMT variants are designed in such a way that pulls for such **proximity errors** (Lezak et al., 2012). Research has shown that normal control subjects can make at least one error on both parts of the TMT (Ruffolo et al., 2000; Lezak et al., 2012) In fact, several factors such as age (Płotek et al., 2014) or educational level (Płotek et al., 2014) and even shift work history (Titova et al., 2016) can affect performance. However, an increased number of errors, especially on TMT-B, has been associated with dorsolateral frontal lobe lesions (Kopp et al., 2015) and this finding has been consistent even when compared to subjects with inferior medial frontal lobe and posterior lobe lesions, who made fewer errors or were completely unaffected (Stuss et al., 2001; Lezak et al., 2012). Chan et al. (2015) challenged the notion that increased number of errors on TMT-B was specific to frontal lesions but did confirm that there was a significant difference in a number of errors when comparing groups with either frontal or non-frontal lesions to healthy controls. Moreover, medial temporal lobe atrophy has been shown to be the strongest neuroanatomical predictor of TMT-B performance in elderly subjects (Oosterman et al., 2010), when analyzed together with periventricular and white matter MRI hyperintensities.

Several variants of the TMT task have been applied as fMRI paradigms. Verbal variant (counting from 1 to 24 or alternate numbers and letters), showed brain activations predominantly in the left frontal lobe hemisphere structures including dorsolateral prefrontal cortex (DLPFC), ventralateral prefrontal cortex (VLPFC) and premotor and motor areas, and in the right hemisphere the cingulate and intraparietal sulci (Moll et al., 2002).

Zakzanis et al. (2005) used MRI compatible writing device called virtual stylus to perform the original visuospatial TMT task. The study elicited the brain activity in three clusters. The first cluster involved activity in frontal lobe areas of the left hemisphere as reported previously by Moll et al. (Moll et al., 2002), including the medial and dorsolateral prefrontal cortex (PFC), precentral gyrus, cingulate gyrus, and insula. The second cluster in the right hemisphere included cingulate cortex and insula. The smallest third cluster included the left middle and superior temporal gyrus suggesting the utilization of internal speech processing. Similar findings suggesting the involvement of PFC, visuomotor and speech processing areas, including Broca's area, have been reported also in functional near-infrared spectroscopy (fNIS) study by Hagen et al. (Hagen et al., 2014). Another TMT adaptation with fMRI event-related design (Allen et al., 2011) was used in healthy participants, who had to visually scan image with a pseudo-randomly distributed array of 22 items and press the button when correct letter/number was localized. Interestingly, this study failed to find PFC activation previously reported in mental set switching. The results highlighted bilateral activations in ventral and dorsal visual streaming and motor response related brain areas.

Few studies have demonstrated the difference between TMT B and A variants (Moll et al., 2002; Jacobson et al., 2011), showing that TMT-B task elicited stronger brain activity in the bilateral DLPFC, right VLPFC and precentral gyrus and left temporoparietal area. Similarly, combined usage of computer version of TMT and fNIS reported blood flow increases in the bilateral PFC when contrasting TMT B vs. TMT A (Kubo et al., 2008).

The number of studies comparing resting state fMRI (rsfMRI) activity and neuropsychological tests performance is sparse. A recent study in healthy volunteers (James et al., 2016) showed a significant effect of the TMT B performance (time to complete) to resting-state connectivity of two regions of interest (ROIs), the right VLPFC and left superior parietal lobule.

**Figure 1:**
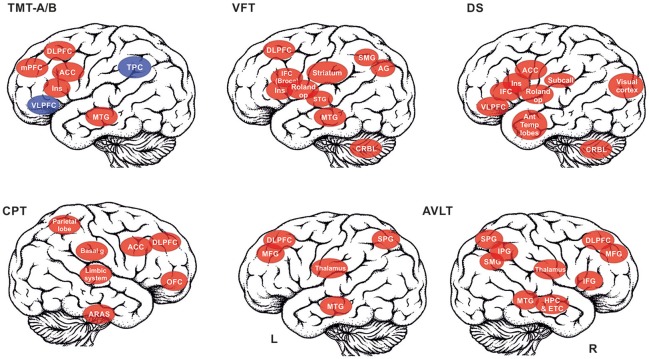
The summary of the brain structures that are recruited in the selected cognitive tests is depicted: TMT A/B, Trail making test, A and B versions (red color for TMT A, red+blue for TMT B); VFT, Verbal fluency test; DS, Digit span; CPT, Continuous performance task; AVLT, Auditory verbal learning test (reported separately for left (L) and right (R) hemisphere). List of abbreviations of brain areas in alphabetical order: ACC, anterior cingulate cortex; AG, angular gyrus, Ant Temp lobes – anterior temporal lobes; ARAS, ascending reticular activation system; Basal g, basal ganglia; DLPFC, dorsolateral prefrontal cortex; CRBL, cerebellum; ETC, entorhinal cortex; HPC, Hippocampus; IFC, interior frontal cortex (e.g., Broca's area); IPG, inferior parietal gyrus; MFG, middle frontal gyrus; mPFC, medial prefrontal cortex; MTG, middle temporal gyrus; OFC, orbitofrontal cortex; Roland op, Rolandic operculum; SMG, supramarginal gyrus; SPG, superior parietal gyrus; STG, superior temporal gyrus; Striatum,– incl. putamen, nucleus caudatus, globus pallidum; Subcall, subcallosum; TPC, temporo-parietal cortex; VLPFC, ventrolateral prefrontal cortex.

The summary of the brain activations is schematically depicted in Figure 1.

### Behavioral TMT performance and brain correlates in schizophrenia patients

Although errors on the TMT are not specific to one psychiatric condition (Moritz et al., 2001), there is evidence that schizophrenia patients show an overall slower processing speed, impaired visuomotor tracking and switching ability (Rodriguez et al., 2015). The studies focusing on the specific TMT errors in schizophrenia are limited, though they have demonstrated that patients with schizophrenia tend to make some errors more consistently. Notably, Mahurin et al. (2006) showed that schizophrenia patients make more sequencing, or as operationally defined in this research “tracking,” errors compared to both depressed patients and healthy controls, which they attributed to a greater degree of cognitive disorganization in schizophrenia.

Imaging studies using TMT task in schizophrenia are sparse. Some studies reported that TMT performance in schizophrenia patients could be predicted by resting state metabolism measured using positron emission tomography (PET) (Horacek et al., 2006) or by resting state connectivity (Argyelan et al., 2013). A recent study using transcranial direct-current stimulation (tDCS) reported altered hemodynamic pattern during the TMT task performance in middle cerebral arteries (Schuepbach et al., 2016).

## Continuous performance test (CPT)^2^

### Behavioral performance (errors) and brain correlates of CPT in healthy and lesion cohorts

In order to test sustained attention and vigilance, many tasks using similar paradigm have been developed. One of the most applied measures is the Continuous performance test (CPT), which assesses four aspects of attention: inattentiveness, impulsivity, sustained attention, and vigilance. The main categories of errors performed in the CPT are omissions and commissions. **Omissions** are made when the respondent does not react to target letters (“non-X”). Results from studies with patients who had damage to the basal ganglia showed more omissions errors (Levin et al., 1986; Wolfe et al., 1990). More omissions errors, longer reaction time and the greatest vigilance decrement are associated with right frontal damage (Rueckert and Grafman, 1996). **Commissions** result from the response to non-target letters (“X”). There are several subtypes of commissions errors: “fast reaction-time response” that is associated with impulsivity and a “slow reaction time response” or “delayed response” as a result of inattention; and a “random” type that is associated with a lack of control (Halperin et al., 1991). **Perseverations** can be also considered as a type of error. The perseverative response in CPT is any reaction time that is less than 100 ms. Such responses are: a slow response to the preceding stimuli, a random response, an anticipatory response, or a response repeated without consideration of the stimuli or task requirements (Conners, 2014).

CPT test is known as a reliable measure of attention (Rosvold et al., 1956; Riccio et al., 2002). Lesion studies show that especially lesions and damages in the right frontal area effect CPT performance. The more severe lesions, the bigger attentional problems, and worse CPT performance has been reported (Katz et al., 1996; Riccio et al., 2002). Riccio et al. (2002) see CPT test as a good symptom-specific measurement but a poor disorder-specific test. They suggested the term “asymmetry” attention and show in their review that the right hemisphere is more activated during the CPT test and that the test is connected with models of attention including cortical (frontal, temporal, parietal), subcortical [limbic, basal ganglia and ascending reticular activating system (ARAS)] and functional systems (pathways between the basal ganglia, thalamus and frontal lobes) (Riccio et al., 2002). Ogg et al. (2008) show correlation between healthy adults' reaction time in Conners' CPT test and anterior cingulate cortex activation. Activation in the right hemisphere was generally correlated with the reaction time. They also point out an extensive network of brain regions associated with visual processing, motor control, and visual attention that is activated during the test, while some areas, such as posterior cingulate gyrus are deactivated (Ogg et al., 2008).

### Behavioral CPT performance and brain correlates in schizophrenia patients

In the CPT test the respondent has to decide whether to respond or not, as well as the maintaining arousal and self-monitoring of behavior (self-control) are needed. Schizophrenia patients are prone to attention/vigilance impairments and self-monitoring dysfunction (e.g., Stirling et al., 1998). A large multi-site study of the Consortium on the Genetics of Schizophrenia (COGS) showed that schizophrenia patients performed poorly compared to healthy subjects, even when controlled for differences in age, sex, education, and racial distribution (Gur et al., 2007). The studies focusing on neurocognitive deficits in schizophrenia and including CPT measures are consistent in their results, showing more commission and omission errors in schizophrenia patients (Earle-Boyer et al., 1991; Elvevag et al., 2000).

First study combining fMRI with CPT task in schizophrenia was performed by Volz et al. (Volz et al., 1999). Systematic review of scientific literature on fMRI studies using a sustained attention task was published by Sepede et al. (Sepede et al., 2014). The review included 11 studies of patients with schizophrenia, of which four studies used the CPT test paradigm: 2 studies used CPT-X (i.e., Eyler et al., 2004; Honey et al., 2005) and 2 studies CPT-IP (i.e., Volz et al., 1999; Salgado-Pineda et al., 2004). A recent imaging study applied an fMRI paradigm of the dual response AX-CPT test version (i.e., Lesh et al., 2013). Significant differences in activation patterns between patient and control groups were found in all studies selected by Sepede et al. (Sepede et al., 2014), even in case the patients performed comparable to the control group (e.g., Eyler et al., 2004). All of the mentioned studies support the finding of the attentional deficit in schizophrenia tested in CPT variants. This deficit was mainly related to hypoactivity in anterior and posterior cingulate cortex and in the right prefrontal cortex (Sepede et al., 2014). Thalamus activation results are inconsistent, reported to be either hyperactivated in SZ patients during CPT (Honey et al., 2005), or hypoactivated (Volz et al., 1999; Salgado-Pineda et al., 2004). Altered activation pattern was also reported in the thalamus. However, both thalamic hypoactivation and hyperactivation have been reported (for more details on imaging studies in schizophrenia see Table 1).

**Table 1:** summarizes the brain correlates and test performance in schizophrenia.

**TASK**	**Study references**	**Task variant**	**Study sample**	**Imaging method**	**Reported brain areas**	**Task-related results and comments**
TMT	Horacek et al., 2006	TMT A and B	SZ (*N* = 42), HC(*N* = 42)	(18) FDG positron emission tomography (PET), TMT A and B performance as covariates.	Higher metabolic activity in temporal, parietal, pre- and postcentral gyri, precuneus, limbic regions (anterior cingulate, uncus) and pons predicted better performance on TMT B (independent of the group).	TMT A and TMT B “time”—SZ (↓↓) vs. HC.
	Argyelan et al., 2013	TMT A	SZ (*N* = 18), BD (*N* = 19), HC(*N* = 32)	Resting state fMRI	Decreased functional connectivity of the left caudate nucleus, temporal occipital fusiform cortex/lingual gyrus, left thalamus predicts worse performance on TMT A in SZ patients (effect gradient: SZ>BD>HC)	TMT A “time”—SZ(↓) and BD vs. HC.
	Schuepbach et al., 2016	TMT A and B	FEP (*N* = 15), HC (*N* = 15)	Functional transcranial Doppler sonography	The blood flow velocity (BFV) measured in middle cerebral arteries during TMT B was significantly increased in patients in comparison to HC. In contrast to patients, BFV of HC subjects returned during TMT to low initial levels.	TMT A and TMT B “time”—SZ (↓↓) vs. HC, “number of errors” (no group differences).
	Liu et al., 2017	TMT A	Adolescent-onset SZ (AOS) (*N* = 48), HC (*N* = 31)	rsfMRI, voxel-mirrored homotopic connectivity (VMHC) and support vector machine (SVM) analyses	AOS group vs. HC exhibited decreased VMHC values in the following brain regions: fusiform gyrus, superior temporal gyrus (STG)/insula, precentral gyrus, and precuneus.	TMT A “time”—SZ (↓) vs. HC Decreased VMHC values in the superior temporal gyrus/insula correlated with slower TMT-A performance.
CPT	Volz et al., 1999	CPT-IP	SZ (*N* = 14), HC (*N* = 20)	fMRI	SZ patients exhibited decreased CBF in the right medial PFC, the right cingulate cortex and the left thalamus in comparison to HC.	CPT-IP performance—SZ vs. HC (no group effect) CPT-IP performance in male subjects was associated with activation pattern (poor performers with SZ showed more active brain regions than HC group and good performers with SZ. Poor performers with SZ vs. poor HC performers decreased dorsolateral PFC activation, increased in right temporal lobe).
	Eyler et al., 2004	CPT-X	Chronic SZ (*N* = 9), HC (*N* = 10)	fMRI	Activation of the frontal cortex was impaired in SZ compared to HC, specifically in the inferior frontal gyrus. These findings confirm the results of (Volz et al., 1999) and (Lesh et al., 2013), however, the localization of the prefrontal deficit differs.	CPT-X performance—SZ vs. HC (no group effect).
	Honey et al., 2005	CPT-X (addition of degraded stimuli to test cognitive dysmetria)	SZ with positive and negative symptoms (*N* = 11), SZ with predominant positive symptoms (*N* = 11), HC (N = 12)	fMRI	SZ group showed abnormally high response to the CPT task in thalamus, left caudate nucleus and in frontal and temporal regions. Contrary, hypoactivation was observed in the middle frontal gyrus, putamen and angular gyrus.	CPT-X performance—SZ vs. HC: “target discrimination” (↓) in SZ, “reaction time” (no group effect) SZ subjects failed to elicit a task-related activation in response to the demands of the degraded stimuli. The pattern of task-related connectivity was disrupted in SZ.
	Lesh et al., 2013	AX-CPT test version	FEP (*N* = 43), HC (*N* = 54)	fMRI	The dorsolateral PFC (DLPFC) activation in FEP was significantly associated with disorganization symptoms and performance during proactive cognitive control measured by CPT [AX-CPT]. SZ vs. HC: reduced recruitment of DLPFC and parietal cortex during CPT.	AX-CPT performance—FEP (↓↓) vs. HC.
VFT	Baaré et al., 1999	Category and letter VFT	SZ (*N* = 14), HC (*N* = 14)	Structural MRI, VBM	Lower semantic fluency scores in SZ patients correlated with smaller volumes of prefrontal gray matter bilaterally.	SZ vs. HC: letter VFT score (↓), semantic VFT score (↓↓) in SZ.
	Sanfilipo et al., 2002	Letter VFT	Male SZ medically stable (*N* = 62), HC (*N* = 27)	High-resolution MRI	Lower VFT scores in SZ correlated with smaller gray matter volumes in the prefrontal and temporal lobes bilaterally.	VFT score—SZ (↓↓) vs. HC Inverse correlations observed between cognitive abilities (psychomotor speed, cognitive flexibility and verbal fluency) and negative symptom.
	Weiss et al., 2004	Letter VFT	High-functioning SZ (*N* = 9), HC (*N* = 9)	fMRI	Bilateral activation inferior frontal gyrus area in a group of SZ patients, in contrast to the activation in left Broca's area seen in HC.	Letter VFT score—SZ vs. HC (no group effect)
	Boksman et al., 2005	Letter VFT	FEP (*N* = 10), HC (*N* = 10)	fMRI	HC group showed activations in the posterior parietal lobe, occipital lobe and cerebellum that were not detected in SZ patients.	Letter VFT “words”—FEP (↓) vs. HC.
	Fu et al., 2005	Letter VFT with 4 different letter sets (2x easy, 2x difficult conditions)	SZ, acute psychosis (*N* = 9), SZ in remission (*N* = 10), HC (*N* = 11)	fMRI	Increasing task demands (difficulty) led to greater anterior cingulate and right middle frontal activation in patients with active psychosis than in patients in remission. Decreased activation in SZ compared to HC in the anterior cingulate and the right prefrontal cortex (inferior and middle frontal cortices) independent of psychotic state and task demands.	VFT “number of errors”—SZ vs. HC (no group effect); in both groups, more errors observed during the difficult condition.
	Weiss et al., 2006	Letter VFT	Unmedicated SZ patients during an acute psychotic episode (*N* = 7)	fMRI	Bilateral activation of inferior frontal gyrus in SZ patients group was associated with the impaired verbal fluency performance.	Letter VFT score—SZ (↓) vs. HC. No evidence of decreased language-related activity in the left hemisphere of the SZ group. Gender differences in Processing strategies for phonemic verbal fluency tests were obtained.
	Takizawa et al., 2008	Letter VFT	SZ (*N* = 55), HC (*N* = 70)	52-channel near-infrared spectroscopy (oxy-hemoglobin concentration)	SZ patients' performance associated with slower and reduced increase in the prefrontal activation as compared to HC.	Letter VFT—SZ (↓↓) vs. HC.
	Bhojraj et al., 2009	Letter VFT and category VFT (20 s trials) from *Multilingual Aphasia Examination: Manual of instructions* (Benton and Hamsher, 1978)—total VFT scores	HC: young healthy adults- high genetic risk for SZ (*N* = 60), no-risk HC (*N* = 42)	MRI surface based volumetry (Freesurfer)	The high-risk subjects had verbal fluency deficits and decreased volumes of gray matter in the left pars triangularis, left supramarginal gyrus and right angular and Heschl's gyri.	High risk subjects vs. no-risk HC: “letter VFT (↓), category VFT (no group effect)”. Left over right hemispheric asymmetry in the pars triangularis of the no-risk HC group (reversed in high risk HC group) suggested as a predictor for improved performance on VFT in both groups.
	Lynall et al., 2010	Letter VFT (FAS version of the Controlled Oral Word Association Test)	SZ (*N* = 12), HC (*N* = 15)	rs-fMRI	In SZ, the reduced degree and clustering were locally significant in medial parietal, premotor, cingulate, and right orbitofrontal cortical nodes of functional networks. Functional connectivity and topological metrics correlated with each other and with behavioral performance on VFT.	Letter VFT—SZ vs. HC (no group effect) When using a DS score as covariate, functional brain networks resulted in the reduced clustering and small-worldness and reduced probability of high degree hubs in the SZ group.
	Meijer et al., 2011	Category VFT	Ultra-high risk (UHR) of psychosis subjects (*N* = 37)	Structural MRI, VBM	Lower VFT scores correlated with lower density in the gray matter of the right superior and middle temporal gyrus, right insula, and left anterior cingulate cortex.	Performance on the VFT may correspond to structural alterations in the brain. No difference in VFT performance between UHR subjects who developed a psychotic illness in the 2-year follow-up and subjects who did not transit to psychosis.
	Vandevelde et al., 2017	Letter VFT	SZ (*N* = 15), BD (*N* = 14), HC(*N* = 20)	fMRI	Study identified three activation clusters. In patients with schizophrenia there was reduced connectivity in a specific medio-prefronto-striato-thalamic network, unlike the bipolar patients and HC group. Suggesting a potential diagnostic significance of this network in SZ.	No group effect in VFT performance. Functional disturbances and different brain connectivity patterns may also be an etiologic factor of poor VFT performance.
DS	Minatogawa-Chang et al., 2009	DS forward and backward	FEP (*N* = 88), HC (*N* = 86)	Structural MRI, VBM	Correlations between the performance in the neuropsychological tests and the GM volume in the DLPFC as well as lateral parietal and superior temporal gyrus.	DS score—SZ vs. HC (group comparison not reported).
	Lynall et al., 2010	DS forward and backward	SZ (*N* = 12), HC (*N* = 15)	rs-fMRI, DS score as covariate The study measured aspects of both FC and functional network topology.	There were no significant associations between any connectivity or topological metrics and either forward or backward DS scores.	DS score—SZ vs. HC (group comparison not reported).
AVLT	Crespo-Facorro et al., 1999	RAVLT A/B recall—recall of both well-learned and novel word lists (A and B list of the Rey AVLT)	SZ (*N* = 14), HC (*N* = 13)	PET	Patients fail to activate cortical-cerebellar-thalamic-cortical circuitry during recall of both well-learned and novel word lists.	RAVLT “practiced and novel conditions”—SZ vs. HC (no group effect)
	Hazlett et al., 2000	CVLT based—errors related activity	Unmedicated SZ (*N* = 20), HC (*N* = 32)	fMRI	Serial-ordering strategy was associated with decreased activity in frontal cortex and increased activity in temporal cortex. Patients also exhibited hypofrontality (lower ratio of frontal to occipital activations) compared with healthy group.	CVLT “number of recalled words”—SZ vs. HC (↓↓). More severe hypofrontality associated with increased perseveration errors in SZ.
	Hofer et al., 2003	Verbal recognition	SZ in remission (*N* = 10), HC (*N* = 10)	fMRI	SZ group exhibited lower activation of right dorsolateral and anterior PFC, right anterior cingulate and left lateral temporal cortex during encoding; less activation in bilateral prefrontal and lateral temporal cortex during recognition.	AVLT “recognition task accuracy”—SZ vs. HC (no group effect).
	Ragland et al., 2004	Verbal encoding	SZ (*N* = 14), HC (*N* = 15)	fMRI	SZ group showed impairment in activation measured by BOLD signal in bilateral prefrontal cortex during encoding of words, but increase activation in parahippocampal gyrus.	AVLT “recognition task accuracy”—SZ (↓↓) vs. HC Words recognition showed decreased activation of the left PFC.
	Allen et al., 2009	Deese-Roediger-McDermott false memory task	At-risk mental state (ARMS) subjects (*N* = 18) HC (*N* = 22)	fMRI	The high-risk group showed decreased activation in the medial temporal cortex and prefrontal regions, during both verbal encoding and recognition. These differences were associated with decreased recognition performance and the increased risk of psychosis.	ARMS vs. HC: “target items accuracy” (↓), lure words accuracy (ns.), “false recognition responses for novel words” (↓) Correct recognition relative to false alarms was associated with differential engagement of the hippocampus bilaterally in HC, this difference was absent in the high-risk group.
	Hurlemann et al., 2008	RAVLT	Early prodromal states (EPS) (*N* = 20), late prodromal states (*N* = 16), HC (*N* = 30)	Structural MRI, VBM	Reduction of hippocampal volumes in late but not early prodromal states correlates with poorer performance in RAVLT delayed recall.	The groups differed only in delayed recall LPS (↓) vs. EPS vs. HC

*Arrows are applied in means of group performance evaluated (independent of the parameter measured in the task) and display significance level of performance (↓ - p < 0.05; ↓↓ - p < 0.01). Abbreviations: FEP, first episode psychosis; SZ, schizophrenia; HC, healthy controls; SA, schizoaffective disorder; BD, bipolar disorder; EPS, early prodromal states; LPS, late prodromal states; DCS, transcranial direct current stimulation; fMRI, functional magnetic resonance imaging; PET, positron emission tomography; VBM, voxel-based morphometry; rsfMRI, resting state functional magnetic resonance imaging; CBF, cerebral blood flow; PFC, prefrontal corte*.

## Verbal fluency (VFT)^3^

### Behavioral performance (errors) and brain correlates of VFT in healthy individuals and in lesion studies

Verbal fluency tests (VFT) require the subject to produce words according to a specific rule and were designed to evaluate several aspects of verbal behavior including cognitive flexibility, switching response sets, self-regulating and self-monitoring (Lezak et al., 2012). Primarily, the VFT assesses higher functions of verbal organization and management (Bertola et al., 2014). The typical two verbal fluency tests defined by Lezak et al. (2012) and Laine (1988) are a category fluency and a letter fluency. In category or semantic fluency test, the subject is required to generate a list of words that are associated with their meaning (e.g. a list of animals or fruits). In letter or phonemic fluency, phonological clusters are made that are either words with the same initial letter or homonyms (e.g., fair, fare) (Lezak et al., 2012). Participants are not allowed to repeat the same word thus indirectly assessing their short-term memory, since they have to remember which words have already been said (Estes, 1974; Lezak et al., 2012; Fischer-Baum et al., 2016). Other significant cognitive functions that the VFT assesses are lexical-semantic knowledge and automatic retrieval (Hurks et al., 2010), controlled information processing (Hurks et al., 2006), sustained attention, strategic planning, searching and inhibition (Birn et al., 2010).

Qualitative errors encountered in a verbal fluency test include **breaking set errors (intrusions)** and **repetition (perseverations) errors**. The latter can be further divided (Galaverna et al., 2016) into simple repetitions, true perseverations and using the same stem in two words (e.g. “paint,” “painter”). **True perseverations** occur in consecutive words whereas simple repetitions are made after a few seconds, possibly showing decreased searching and inhibition skills or even deficits in working memory (Azuma, 2004; Lezak et al., 2012; Fischer-Baum et al., 2016). **The intrusions or breaking set errors** refer to inappropriate responses with production of words with a different initial letter or different category than the assigned one (Lezak et al., 2012; Galaverna et al., 2016). Intrusions are associated with decreased inhibition and/or increased susceptibility to interference (Mahone et al., 2001).

Chertkow and Bub (1990) noted that generating words beginning from the same letter is not practiced as a skill in everyday life thus requiring strategic thinking whereas category fluency is based on “conceptual knowledge.” This may be one of the reasons why category fluency scores are overall better than letter fluency, even in healthy control groups (Laws et al., 2010). Several studies have shown that increasing age and lower education level strongly correlate with poorer performance on category fluency, both in terms of volume of words generated and number of errors made (Mitrushina et al., 2005; Lezak et al., 2012). Importantly, Weiss et al. (2003) showed that, when controlling for performance differences, males and females show the same brain activation pattern during the verbal fluency task.

Shao et al. (2014) evaluated specific skills required for the VFT, including vocabulary knowledge, lexical access speed and executive control ability and how they correlate with category and letter fluency scores. Vocabulary knowledge and lexical access speed were shown to be more predictive of the category fluency performance compared to the letter fluency, while executive control ability did not have a significant effect on one variant over the other (Shao et al., 2014). The distinct aspects of cognitive performance that the VFT examines are further verified by studies showing that different brain regions are involved in letter and category fluency. In a meta-analysis of 31 studies, Henry and Crawford (2004) compared patients with focal cortical lesions to healthy controls. They showed that temporal lobe damage correlated with poorer performance on category fluency tasks whereas lesions in the frontal lobe negatively affected both category and letter fluency to the same extent (Henry and Crawford, 2004). Particularly left inferior frontal lobe was repeatedly demonstrated to be active to a various extend in both VFT variants [meta-analysis and systematic reviews by Costafreda et al. (2006) and Wagner et al. (2014)]. A recent study highlighted the important role of the basal ganglia in both letter and category fluency. Chouiter et al. (2016) examined a group of 191 right-handed patients who had suffered a first, unilateral, focal lesion either in the left or right hemisphere. Results showed that letter and category fluency had certain identical regional associations in the left hemisphere, namely putamen, caudate nucleus, globus pallidum, superior and middle temporal gyri, angular gyrus, insula and parts of the supramarginal gyri, supporting the notion of a common word-producing mechanism (Chouiter et al., 2016). Additionally, letter fluency performance correlated with lesions in the rolandic operculum and the supramarginal gyrus unlike category fluency, which was preferentially affected by lesions in the posterior middle temporal gyrus and pallidum (Chouiter et al., 2016). Marien et al. (2001) proposed a significant role of the right cerebellum in retrieval and other non-motor language aspects, observing the significant linguistic deficits in patients with lesions in this area.

### Behavioral performance and brain correlates of VFT in schizophrenia

Deficits in verbal fluency in schizophrenia patients are not a surprising finding but there are two competing theories on whether they should be attributed to a diminished access to the semantic store (Joyce et al., 1996) or to a disrupted semantic store (McKay et al., 1996). In the former case, category and letter fluency scores should be equally affected whereas, according to the latter theory, category fluency performance would be lower than letter fluency, since it is more dependent on semantic memory. Henry and Crawford (2005) conducted a large meta-analysis on 84 studies, comparing verbal fluency scores between schizophrenia patients and healthy controls, and found that deficits in category fluency are more pronounced compared to letter fluency, thus supporting the concept of a compromised semantic store despite generally lower retrieval ability. They also proposed that category fluency can be a predictive test in estimating the probability of future psychosis development.

Volumetric differences in specific brain areas, such as gray matter volume in the prefrontal and temporal lobes, related to altered VFT performance in schizophrenia patients have been documented using both voxel-based morphometry (VBM) by Meijer et al. (2011) and high resolution MRI methods (Baaré et al., 1999; Sanfilipo et al., 2002). Functional disturbances and different brain connectivity patterns might also present an etiologic factor of poor VFT performance (for more details see Table 1). Several fMRI studies have indicated decreased activity in the right anterior cingulate cortex, prefrontal, inferior frontal and middle frontal lobe in patients with schizophrenia undergoing verbal fluency tasks (Boksman et al., 2005; Fu et al., 2005).

## Digit span (DS)^4^

### Behavioral performance and brain correlates of DS in healthy and lesion cohorts

The Digit Span test (DS), which is part of the Wechsler batteries (the intelligence and memory scales), is widely used for measuring of the immediate and working memory. Beside standard performance scores, the errors that are possible to detect in DS, can be divided into two categories: **item errors** and **order errors**. In item errors there is a change in the length of the span. In order errors, there is a change in the order of the digits in the sequence but the length of the sequence remains the same. In the item error category, one can distinguish two **types of errors: omission and insertion**. These types of errors occur at the end of the span length (in DSF they occur at the beginning and in DSB in the end). They are also more common for longer sequences (Woods et al., 2011). **Omission** errors occur when a subject fails to repeat one or more digits of the sequence, while the rest of the span is in a correct order. Kaplan (1991) suggested that an omission in shorter span can be a result of attention shifting, while an omission in longer span might signify a true memory deficit. An **insertion** error is the addition of an extra digit, which results in a longer span. In the order error category, there can be 3 different types of errors: sequencing, substitution, and repetition. These types of errors occur usually in the middle of the span and are more common for spans of shorter digits (Woods et al., 2011). **Sequence errors** present as a span with the correct length but a part of the span is in incorrect order (e.g., 1367 à 1637). **Substitution errors** occur when a subject replaces one digit for another, that could be part of the sequence or not (e.g., 23,578 and 23,478); here again, the span has the right length. **A repetition error** is the duplication of a digit, that appears in the same span (Woods et al., 2011).

The various MRI studies have helped to identify the brain activity associated with the performance of the DS. As demonstrated by Taki et al. (2011), the performance of DG positively correlated with the percentage of gray matter volume in the intracranial volume in the bilateral anterior temporal lobes. Another study that utilized voxel-based morphometry (VBM) and functional connectivity measures, confirmed the bilateral activation of anterior temporal lobes together with the left inferior frontal gyrus and the left Rolandic operculum, which constitute the critical areas in the auditory phonological loop of the verbal working memory (Goldman-Rakic, 1996). Along with the structural findings, DS scores were positively correlated with the resting state networks (rsN), namely the salience network (SN), that is, between the right anterior STG, the dorsal anterior cingulate cortex and the right fronto-insular cortex. It anti-correlated with the resting state functional connectivity (rsFC) within an anti-correlation network of the SN, between the right posterior superior temporal gyrus and the left posterior insula. Authors suggested such pattern of the activation reflected the neural organization of the phonological loop (Goldman-Rakic, 1996).

Another study demonstrated age-related and independent brain correlates with DS performance (Yang et al., 2015), specifically dorsal anterior cingulate gyrus showed distinctive roles in forward and backward span, whereas age dependent structures of the angular gyrus and sub-callosum were associated with DSF performance, and visual cortex and VLPFC were linked to DSB performance depending on age.

Lesion studies contribute to the understanding of the neural processes during DS performance challenging previously hypothesized neural targets. A study by Cave and Squire (Cave and Squire, 1992) showed in a sample of amnestic patients with hippocampal lesions and those with Korsakoff syndrome with a diencephalic damage that the DS scores of amnestic patients were performing close to controls, while the scores were significantly lower in the Korsakoff syndrome group. These findings support the view that the deficit in performance is independent of the hippocampal function. Another study challenged the role of the cerebellum in DS processing. In a single case study, a patient with a bilateral cerebellar ischemic lesion showed preserved DS performance (Chiricozzi et al., 2008).

### Behavioral DS performance and brain correlates in schizophrenia patients

Behavioral studies utilizing the digit span test have repeatedly shown a significantly impaired performance in individuals suffering from schizophrenia when compared to the healthy controls (Haenschel et al., 2009; Park and Gooding, 2014). In DSF the differences are controversial since some studies have shown that there are differences in span length between patients and healthy controls (Conklin et al., 2000; Galaverna et al., 2012) whereas others demonstrated similar performance in both groups (Moritz et al., 2001; Frydecka et al., 2016), suggesting the DSF test is not as demanding as DSB. On the other hand, the differences in DSB between patient and controls are quite significant. Several studies assessing working memory in individuals with schizophrenia using DS, have shown that not only the patients (Brébion et al., 2009) but also their first-degree relatives are able to remember shorter spans than the healthy controls (Conklin et al., 2000; Park and Gooding, 2014), which shows that working memory impairment is schizophrenia can have an endophenotypic character.

Studies reporting morphological or functional alterations associated with poor DS performance in schizophrenia are surprisingly rare (see Table 1). Minatogawa-Chang et al. (2009) reported significant correlations between the performance in DS task and the gray matter (GM) volume of DLPFC, parietal and temporal regions in first-episode psychosis (FEP) patients and healthy subjects. Interestingly, the middle frontal gyrus (BA46) GM volume was correlated only with the performance in FEP. On the other hand, the study by Lynall et al. (2010) failed to find any association between functional alterations in connectivity patterns measured during resting state and DS performance of schizophrenia patients. Studies reporting qualitative analyses of DS-related errors in schizophrenia and its association with functional or morphological changes are completely missing.

## Auditory verbal learning task (AVLT)^5^

### Behavioral performance (errors) and brain correlates of AVLT in healthy individuals and in lesion studies

One of the most often applied learning and memory tests is the Rey Auditory-Verbal Learning Test (RAVLT) with a list containing 15 semantically unrelated words, contrary to the other AVLT variant, the California Verbal Learning Test (CVLT), which includes 16 semantically-related words (Mitrushina et al., 2005)^5^. The RAVLT method is very popular among clinicians for a good reason, as it allows to separate individual memory processes that could be responsible for the identified disturbances of learning and memory.

The most commonly used measure in AVLT is the total number of correct responses (T_1−5_)^6^ that informs us about the immediate recall score and, in terms of repeated trials, about the learning curve. Complete list of possible performance scores that can be calculated in RAVLT are well documented in Bezdicek et al. (2014). Here we will mention only the error-related qualitative approaches. The analysis of errors provides information about the memory processes and their integrity. Usually, only errors made during five consecutive learning trials (T1–T5) are reported (see e.g., Schmidt, 1996; Preiss et al., 2012). However, the quality of individual errors both during recall and recognition trials can also be recorded and analyzed. Two type of errors are usually detected: **Repetition errors or perseverations** are counted if the same correct word is listed more than once during one recall trial (recurring words), and are an important sign of impaired self-monitoring function (Lezak et al., 2012)^7^. **Intrusion errors** (confabulations or false productions) bring us more detailed information about the memory processes. Schnider et al. (1996) suggests that intrusions partially reflect the process of the effortful retrieval of memories despite the weak memory trace. Cunningham et al. (1997) suggested utilization of the so called “confabulation index” for quantification of confabulations in research studies, calculated as the proportion of novel recall intrusions to total responses. It is, however, important to distinguish provoked and spontaneous intrusions. Provoked intrusions from the list A to the interference list B or from the interference list B to post-interference recall of list A indicate sensitivity toward proactive interference and weakness of the context memory as suggested by Geffen et al. (1990). According to Barba et al. (2002), a weak memory trace may be a prerequisite for the occurrence of intrusions (promoted by interference at encoding). On the other hand, the extra-list intrusions (non-related or spontaneous intrusions) may be treated as a form of confabulation. Such intrusions may also reveal tendencies for semantic (category) or phonetic confusion of the original words (Mitrushina et al., 2005). Intrusion errors may thus serve as a measure of impaired executive functions applied in memory processes and should be analyzed in more detail, in order to prevent the vague interpretation produced by grouping all confabulations together (Cunningham et al., 1997). False recollections (increased number of intrusions) have been together with low recall performance previously described as a pattern typical for patients with focal frontal lobe lesions (Baldo et al., 2002) and dementia with prominent frontal lobe semiology (see Rouleau et al., 2001). Several studies also reported that false positives in recognition tests and intrusions on free recall trials are increased in confabulating patients (Bigler et al., 1989; DeLuca, 1993; Fischer et al., 1995; Cunningham et al., 1997). On the other hand, Nahum et al. (2012) shows that intrusions in memory tests have no association with behaviorally spontaneous confabulations or disorientation.

Brain areas responsible for learning and memory as measured by verbal learning tasks, such as the RAVLT/CVLT, involve mainly frontal and temporal lobe areas, supported both by lesion and imaging studies (Savage et al., 2001; Baldo et al., 2002). Lesion studies, namely, Schouten et al. (2009) demonstrated that poor verbal memory performance as a result of the performance on both immediate and delayed recall and recognition in RAVLT^8^ could be predicted by lesion characteristics. In their study patients with left hemispheric lesions, subcortical and large lesions performed poorly on the verbal memory measures. Medial temporal lobe (MTL) volume predicted the rate of learning in RAVLT in healthy volunteers as well (Fernaeus et al., 2013). Bilateral involvement of frontotemporal areas was also observed in studies that applied AVLT-based verbal memory fMRI paradigms. Johnson et al. (2001) provided evidence of the right frontal and left MTL involvement in verbal memory during CVLT task and documented a positive correlation between the activation of this network and task performance.

In terms of individual AVLT measures, the RAVLT first recall (Trial I) demonstrated the inferior parietal, middle frontal, and temporal activation (Wolk and Dickerson, 2011). Lezak et al. (2012) reported the involvement of MTL in last recall Trial V and hippocampal involvement during a delayed recall. More specifically, the head of the hippocampus is involved in verbal memory tasks (Hackert et al., 2002). Johnson et al. (2001) report the additional involvement of right anterior hippocampus. Recognition scores have been previously associated with the volume of perirhinal and entorhinal cortices Lezak et al. (2012) and right DLPFC activity, particularly in subjects with better memory abilities (Johnson et al., 2001). In healthy controls, the task of recalling the original list of 15-items after 24 h compared to resting baseline showed activation in frontal (left superior, and bilateral inferior and middle frontal gyrus) and parietal cortex (superior parietal gyrus bilaterally, right supramarginal gyrus) (Mensebach et al., 2009).

While the majority of studies focused on the exploration of cortical and hippocampal areas, other cortical and subcortical structures contribute to verbal memory too. Resting state functional connectivity (rsFC) study in a healthy population sample (Ystad et al., 2010) identified the correlations between CVLT measures and thalamic FC^9^. Another study on healthy subjects with memory complaints found RAVLT measures to be associated with glucose metabolism in posterior cingulate, precuneus, and orbitofrontal cortex (Brugnolo et al., 2014).

### Behavioral performance and brain correlates of AVLT in schizophrenia

Verbal learning and memory deficits measured by AVLT tasks (related to frontotemporal dysfunction) have been repeatedly reported both in first episode schizophrenia subjects (FES) (González-Blanch et al., 2007; Pérez-Iglesias et al., 2010; Rodriguez et al., 2015) and chronic schizophrenia patients (for review see Aleman et al., 1999; Boyer et al., 2007) and are considered as some of the main characteristics of cognitive deficits in schizophrenia (Keefe, 2008). One study assessing prodromal states of schizophrenia reported that reduction of hippocampal volumes in late but not early prodromal states correlates with poorer performance in RAVLT delayed recall (Hurlemann et al., 2008).

Even though the study sample of schizophrenia spectrum disorders often presents with a mixture of diagnoses, deficits in RAVLT performance may be a common denominator of the illness, as they are present in both paranoid and undifferentiated schizophrenia subtypes (Seltzer et al., 1997). The RAVLT performance is affected both in drug-free patients and patients on antipsychotic medication and is inversely correlated with negative symptoms (Manglam and Das, 2013), while no association with positive symptoms is observed. In contrary to CVLT, the RAVLT has been selected as a sensitive measure of outcome in schizophrenia (see Lepage et al., 2014) based on findings of several studies that failed to show differences in verbal memory between groups of patients with a different outcome (remitted vs. non-remitted) using CVLT. This could be due to semantically-related words that might help during the encoding process as was suggested by Lepage et al. (2014).

Despite the growing number of studies assessing verbal memory in schizophrenia using RAVLT, most of these studies only report total recall score (Trial 1–5; e.g., in Karilampi et al., 2007) and/or delayed recall performance in T7 (Pérez-Iglesias et al., 2010), while a minority of them report performance measured in particular trials (T1, T5, T6 for retention and recognition trial; e.g., Hurlemann et al., 2008). Specific characteristics of the RAVLT performance, such as errors, are often omitted in reported studies completely. Despite this lack of relevant RAVLT error-related literature, patients with schizophrenia show a higher total number of intrusions not affected by age, sex but correlating with patient IQ (Badcock et al., 2011). Our study performed in a group of FES patients showed an increased number of repetitions but not confabulations (intrusions) in comparison to a group of matched healthy volunteers (Rodriguez et al., 2015).

It has been suggested that errors in general or source memory deficits (repetitions of the correct answers and intra- and extra-list intrusions) underlie the positive symptoms of schizophrenia (Frith, 1995; Brébion et al., 2008, 2009). Some authors observed an association between the global number of extra- and intra-list intrusions and the positive symptoms score (Moritz et al., 2001) or thought disorder (Subotnik et al., 2006). In addition, the tendency to make false recognitions of non-target words may reflect a reality monitoring deficit associated with delusions and thought disorder as suggested by Ragland et al. (2003), and with hallucinations as reported in other studies (Brébion et al., 1998, 2005). In contrast, an inverse association was observed between the global number of intrusions and the negative symptom score (Heinrichs and Vaz, 2004) that might result from intensification of inhibitory processes that prevent intrusions. The higher number of extra-list intrusions during the free recall was negatively associated with certain negative symptoms, such as anhedonia, lack of spontaneity and emotional withdrawal (Brébion et al., 2002). Similar negative correlation with affective flattening was reported by Turetsky et al. (2002). As reported previously, mostly frontotemporal and subcortical networks are active during the performance in AVLT. These same networks are impaired in patients with schizophrenia. Several studies report functional or morphological changes in the medial temporal lobe and/or prefrontal areas related to observed disturbances in the AVLT performance (for more details see Table 1). While PFC activation was mostly reported only during encoding process (Hofer et al., 2003; Ragland et al., 2004), an error-related analysis in the study by Hazlett et al. (2000) showed that perseveration errors in schizophrenia are associated with hypoactivation of frontal areas.

## General discussion

### Theoretical modeling of generalized cognitive dysfunction in schizophrenia by classifying errors and underlying brain mechanisms

Standardized cognitive tests that are applied in schizophrenia research usually provide overall scores for individual tests, neglecting their qualitative characteristics. The test performance in healthy individuals, directly or indirectly supported by brain imaging data suggests the recruitment of the various cortical and subcortical brain structures, which also points to the compounded processing. Oftentimes, in order to explain the complex brain response, we find the explanation that proposes the involvement of various cognitive processes such as attention, memory executive functioning being themselves fairly complex. To date, there are no studies that would match the individual performances with the subject's brain activity, though this could be a desirable approach in clarifying the picture. Furthermore, the studies that are currently present are discrepant, each identifying novel brain structures involved during performance in particular cognitive task. Such a discrepancy in identified brain areas might be explained either by the heterogeneity of the tests' execution among patients or by the improvement of imaging techniques and statistical analysis. Whichever is true, a lack of a strong theoretical framework is obviously a disadvantage in the cognitive neuroscience of schizophrenia.

The attempt of modeling a cognitive dysfunction in schizophrenia proposed by Silverstein (2008) was based on the definition of specific and generalized cognitive dysfunction and was implemented in the large-scale schizophrenia research project CNTRICS. Since the neuropsychological tests are generally confounded by the multiple processes and a number of factors can impact cognitive functioning (fatigue, lack of motivation), in order to address specific deficits, Silverstein suggested: (1) to use a match task approach (application of two tasks that match on the variance and reliability) and (2) to apply process-specific task with the subsequent analysis of the changes across multiple conditions and multiple time points in order. Being complementary, the first approach would help to identify the specific deficits on the behavioral level, while the second—would help building up a mathematical model by application of the analysis of the covariance, principal component analysis, aggregation of scores into the cognitive subdomains, partially ordered sets and process-oriented strategies.

Indeed, the modeling approach has been widely used in cognitive psychology proposing the models for elementary cognitive processing including the reaction times (Townsend and Ashby, 1983) and more complex processing such as memory and reinforcement learning in healthy and clinical populations (Neufeld, 2007, 2015). Several studies aimed at mathematical modeling of the specific cognitive task on learning and memory [e.g., Continuous Presentation Task by Atkinson et al. (1967)], including the cognitive neuroimaging approaches in assessing the process of the decision making (Ahn et al., 2011; White et al., 2012).

Though Silverstein called for the specification of the deficits in schizophrenia, the mechanisms of the generalized deficits (possibly specific for some clusters of schizophrenia patients) seem to be overlooked. The approach based on the analysis of the similar errors that may occur in cognitive tests would allow to identify the common denominators of the generalized deficits. In the current review, we have gathered the characteristics of qualitative performance (errors) that can be detected in commonly used neuropsychological tests. From the summary provided, the similarities and differences in the errors across tests can be identified. For example, in the variety of tests, one can detect **perseverative errors or repetition errors** that are defined as the immediate inappropriate repetition of a prior response and are common for dorsolateral prefrontal cortex and basal ganglia dysfunction (Schindler et al., 1984; Hauser, 1999; Nys et al., 2006). However, Ramage et al. (1999) have shown that about four percent of the healthy cohort with both young and older subjects commit perseverations. Indeed, in children perseverations are normal and are attributed to the brain immaturity and lack of inhibitory mechanisms (Hauser, 1999). The development of the prefrontal cortex can reduce perseverations by supporting the strengthening of active representations in a competition between latent memory traces for previously relevant information and active memory traces for current information (Munakata et al., 2003). **Intrusion errors (form of confabulations)** refer to the inappropriate repetition of prior responses after intervening stimuli (Lorente-Rovira et al., 2011). Schindler et al. proposed that spontaneous confabulations (unprovoked) might be a result of a disconnection between orbitofrontal cortex (through the dorsomedial nucleus) with the amygdala (Schindler et al., 1984). Lesion studies suggest that confabulations are associated with damage in the right ventromedial frontal lobes, cingulate gyrus, cingulum, anterior hypothalamus, and head of the caudate nucleus (Moscovitch and Melo, 1997) and, similarly to perseverations, are detectable in healthy subjects (Burgess, 1996).

Further, **omission errors** correspond to the missing target; and **commission errors** imply the response to any stimulus other than the target as suggested by the instruction; those are typically detected in various GO/noGO task. In the study of Menon and Uddin (2010), the left and right insula and adjoining inferior frontal cortex, right anterior cingulate, and left precuneus/posterior cingulate showed significantly greater activation during error processing (omission), compared to response inhibition (commission) and competition.

Lastly, sequential processing refers to the mental integration of the stimuli in a particular serial order. **Sequential errors** are presumably dependent on the cognitive domain (perceptual, motor). Thus, in the motor system, the underlying pattern of activation involves the primary motor and sensory areas, cerebellum, and basal ganglia (Ghilardi et al., 2009). Also, the sequential errors can be common in phonological processing (Whitaker, 1972). For example, Kuchinke et al. (2009) demonstrated that semantic processing of sequential relations additionally activated left medial and middle frontal gyrus, and left inferior frontal gyrus (Kuchinke et al., 2009). Therefore, each of the types of errors seem to have the unique pattern of the brain activity.

Importantly, besides generating performance errors, the human brain employs a meta-function aimed at monitoring the errors. This prefrontal monitoring system has been studied extensively, with its' center proposed in the anterior cingulate cortex (ACC). ACC is known to serve cognitive control functions enabling the brain to adapt the behavior in accordance to the changing task demands as well as the environmental circumstances (Botvinick et al., 2001).

Given the results of studies reported above, one can hypothesize that the brain encompasses the error detection system that prevents the errors occurrence and keeps monitoring the ongoing performance. Both the failure of error monitoring system and the errors described above can be detected across multiple tests indicating the generalized deficits that has been repeatedly reported in patients with schizophrenia (Goldstein and Shemansky, 1995). In other words, for instance, perseverations could be detected in several tests (not necessarily similar tests) that are predisposed to this type of errors. With respect to the brain activations that are associated with the specific errors, the pattern seems to lie in the central hubs of the cortex (DLPFC, OFC, ACC, PCC, precuneus) or within large scale networks (the description is below) with projections into subcortical structures (basal ganglia, amygdala). In line with error analysis, current evidence suggests an existence of a multiple error processing network in the brain, involving frontal and parietal regions and specifically ACC (Stevens et al., 2009). The structures are usually involved in the successful performance but the degree of activation fluctuates when the error occurs. From the experiment conducted, Stevens et al. also conclude that adults show a greater response amplitude in several error-related networks in comparison to adolescents suggesting that the normal maturation implements the greater responsiveness of the relevant brain structures to errors. Another study of Wierenga et al. (2015) suggested that the connectivity of the unimodal regions strengthens in childhood, while in adolescence the largest changes occur within and between frontal and parietal lobes, presumably indicating the greater flexibility of these regions. Indeed, it seems that the circuits require to sustain a certain level of activity in order to prevent the commission of the errors.

From the summary of the studies provided above, the cognitive test performance is not limited to error detection and “prevention” circuits. From our review of the task performance studies, it seems that many additional probably task/function-related structures are activated during the task performance. Moreover, the subsequent analysis in schizophrenia studies has revealed a diverse constellation of brain structures that are activated during the test performance or when correlating with cognitive performance (distinct from healthy individuals). How can this data discrepancy be interpreted? Firstly, assuming structural and functional brain alterations in schizophrenia, cognitive processing becomes more effortful creating the necessity of additional circuits to be involved. Secondly, in all scrutinized tests, patients exhibited a decreased activity in the medial temporal gyrus or superior temporal gyrus (Goldman et al., 2008). This finding is common in schizophrenia, and may not be related to the test performance *per se*, but rather indicate the group-specific and possibly symptoms related alterations. On the other hand, the alterations in cortical structures, parts of the associative cortex (temporal, parietal, occipital) could contribute to the specific cognitive deficits. The immense variability of the results (activation patterns) obtained from task-related performance studies therefore suggests more general error-related approach as more useful to model cognitive dysfunction in schizophrenia.

### Optimizing the research strategies of brain network analysis

MRI and related methods have been prolific in the identification of networks that may be associated with specific functions. Thus, in the resting state, the cerebral cortex produces consistent spatiotemporal patterns of activity (Damoiseaux et al., 2006). These spontaneously emerging fluctuations map the cortex in a similar way as they are produced during task performance (Deco et al., 2013) being in a “stand-by mode” and indicating the readiness of the system to respond to stimuli (Van Vreeswijk et al., 1994). Resting-state networks (RSNs) are slightly discrepant across different studies (Lowe et al., 1998; Cordes et al., 2001; Damoiseaux et al., 2006), though some studies classify the networks according to their functional role in cognitive processing. The large-scale networks include a salience network (SN), DMN and central executive network (CEN) each of which correspond to a specific functional role, though being functionally linked with each other. The SN involves the dorsal-anterior cingulate and anterior insula regions and is involved in the selection of salient external and interceptive signals (Sridharan et al., 2008). The DMN consists of midline structures, notably the medio-frontal cortex and posterior cingulate, being dominant in the resting state and deactivating during focused activity (van Buuren et al., 2010). The CEN includes the regions in the middle and inferior prefrontal and parietal cortices that are engaged in many higher-level cognitive tasks (Menon, 2011). In the task-related activity, these networks act consensually; CEN activates while being triggered by the externally oriented stimuli while DMN shows decreased activation. The SN causally influences the activation of DMN/CEN by switching between these two networks (Nekovarova et al., 2014). Another classification of functional network was proposed by Power et al. who divided the networks into “processing” or “control” categories (Power et al., 2011). Processing-type networks are static and modular, and control networks are dynamic and adaptable to various tasks. Furthermore, frontal-parietal networks (FPN) that include the lateral prefrontal cortex and posterior parietal cortex presumably play a role in the top-down control (Dosenbach et al., 2008). One more classification of task-positive and task negative networks has been proposed (Fox et al., 2009). It is based on the assumption that the activity within the specific networks correlates positively or negatively based on analogous or opposite functional roles. Task-positive and negative networks are constituted of a set of regions that repeatedly increase-decrease in activity during attention-demanding stats (Fox et al., 2009).

Currently, different approaches for identifying patterns of coherent activity are used for the analysis of resting state networks (for a review see Cole et al., 2010). Functional and effective connectivity are concepts critical to this framework. Seed-to-voxel connectivity approach (Van Dijk et al., 2010) assists in identifying the specific connections that might be attributed to the type of errors in the cognitive tests. Given the fact that the specific type of errors can be detected across the tests (for example sequential errors in a Trail Making Test, Digit Span etc.), this connectivity approach might help to identify the associated brain connections.

A clustering-approach, based on single-subject independent component analysis (ICA) has been introduced by Esposito et al. (2005). The algorithm introduces a complex similarity measure by taking into account spatial and temporal characteristics for clustering. As temporal RSN patterns do not imply very diverse temporal characteristics this leads to unpredictable outcomes.

Functional connectivity fMRI explores correlations between time series from one region of interest (ROI) to another but cannot make inferences about influences between these regions. One can estimate causality of direction between activation in one brain node and another one with the methods based on effective connectivity. The most used means to estimate effective connectivity are Structure Equation modeling (SEM), Psychophysiological interactions (PPI) and Granger Causality (GC)—(can make inferences about linear states) (for review see Stephan and Friston, 2010). There are several differences between these approaches. The Granger Causality models the dependence among observed neural responses or patterns of activity (Friston et al., 2013). The Graph theory envisages the brain as a networked system composed of nodes and links. The various brain sites and anatomical tracts connecting them and their interrelations are encoded in the measures of the statistical dependencies. The networks architecture presumes the existence of the neural hubs, referring to the brain areas that are localized centrally and are densely connected with the other structures, together constituting a “rich club” (van den Heuvel and Sporns, 2013). The rich club networks are also referred to as large-scale networks, notably DMN, CEN and salience network. These networks hold long-range connections between the distant brain areas. On the other hand, the “small-world” networks consist of dense local clusters of connections between neighboring nodes and have a short path length between distant pair of nodes, at the same time being more specialized in function. Over the past years, several reports have consistently suggested that brain hubs and their rich club connections imply the efficient neural communication and integration, constituting “a central communication backbone that boosts the functional repertoire of the system” (van den Heuvel and Sporns, 2013). Schizophrenia patients exhibit the reduced connectivity between the rich club nodes of the brain as well as the small world nets. The same patterns are also present in siblings of patients and in their healthy offsprings (van den Heuvel and Sporns, 2013). Using the information on the networks from the previous step, the number and the properties of connections (centrality, assortativity, transitivity and path length etc.) within and between the defined networks using graph theoretical approach can be explored.

Patients with schizophrenia tend to have a less integrated functional brain connectivity (Lynall et al., 2010). However, the current status quo misses the aspect of the interactions between and within the cognitive functions and brain circuits. In the early review, Pantelis and Brewer (1996) provide an example of the study that tapped into specific errors in the performance. Thus, Owen et al. (1990) have decomposed the analysis of the set-shifting task (WCST) based on the failure in set-shifting or perseverations. This strategy has helped to identify the brain circuits associated with set-shifting (cortical-subcortical axis, basal ganglia) and perseveration errors (frontal), respectively. In this sense, they proposed a dichotomy between component-specific (cortical-subcortical networks that can be prompted by decomposing the complex cognitive functions) and network-specific (frontal-striatal-thalamic circuits coupled with the specific/solid pattern of function or behavior) brain functions. Twenty years later, Sheffield and Barch (2016) highlight the circuits that could serve as a reference point to study cognitive deficits in schizophrenia: (a) task-positive and task-negative functional brain (DMN, frontal-parietal, cingulo-opercular networks); (b) Cortico-Cerebellar-Thalamic-Cortical Circuit (CCTCC) to support main cognitive abilities (DMN, frontal-parietal, cingulo-opercular networks, subcortical networks and cerebellum) (c) the Go/NoGo pathway of reinforcement learning (encompassing the CCTCC networks+activation in the striatum). They point out the necessity to examine interactions between systems in schizophrenia, since the complexity and a range of dysfunctions are hardly due to single system impairments (Barch and Ceaser, 2012). Referring to the recent literature on connectomics (Park and Friston, 2013), it is less likely that only one part of the brain could be responsible for errors during performance. Rather, there is a probability of specific networks impairment. Although it is difficult to derive specific networks from studies above, we can see several areas of the brain that could serve as hubs for these networks.

### Limitations

Several limitations of the study should be mentioned. Firstly, the temporal characteristics of the cognitive processing is not discussed in the review, though it can impact greatly the actual outcome of the brain analysis (Smith et al., 2012). Large scale networks that encompass long-distance projections usually create a faster dynamic and are easily detected in contrast to the local networks (Shaposhnyk and Villa, 2012). However, the local processes presumably occur in the shorter time windows and might be problematic for detection with the current MRI technology. The interactions between the systems are also not mentioned in the current review though the experimental evidence is limited and does not allow to make any predictions (Wang et al., 2016; Dixon et al., 2018; Senden et al., 2018). Moreover, the application of simultaneous EEG-fMRI might be beneficial since to date no studies of this kind exist. In addition, the use of Granger causality methods could be problematic in fMRI as the hemodynamic response function is different between brain areas. This could be confounder in the temporal precedence of neuronal events as well as lower sampling rates or noise (Bajaj et al., 2016). While method is dependent on the selected model there is risk of spurious influence in eluding region, that drives the interactions in the model. The basic model of dynamic causal modeling (DCM) was enriched of modeling of neuronal fluctuations, called the stochastic modeling or spectral models. Thus, DCM could be used also for resting state fMRI data (Frässle et al., 2018). The debate is whether model involves all possible biological knowledge to model neuronal function. Some authors suggest that for example activity dependent plasticity or back-propagation is neglected in standard DCM (Daunizeau et al., 2011) But, Daunizeau also questioned if these specific “fine grained” mechanisms could be captured in BOLD signal. The other limitation of DCM is that the model is limited to maximum 10 regions, though new regression DCM method could possibly extend to whole-brain connectome analysis (Frässle et al., 2018).

Secondly, the selection of the proposed cognitive tasks was driven by the following arguments: (1) all the tasks are a part of the routine neuropsychological examination and are incorporated into the majority of the cognitive batteries used in the cognitive assessment of the patients with schizophrenia; (2) the tests do not explicitly tap into decision making process, requiring only simple manipulation with numbers, letters or words without making a choice (except for CPT test assessing also basic inhibition processes); (3) it was possible to dissect the tests based on the specific types of errors and to track common errors across the tests which served for the modeling purposes. Cognitive mechanisms or cognitive models of other cognitive tasks [for instance, Go/No-Go task (Yechiam et al., 2006)], Iowa Gambling Task (Fridberg et al., 2010), Wisconsin Card Sorting test (Bishara et al., 2010) or Stroop task (Taylor et al., 2016) should be considered in the future analysis.

## Conclusion

Investigation of interconnections between brain networks and cognitive functioning referring to cognitive deficits in schizophrenia on different levels (behavioral (cognitive performance) and physiological (brain networks) of disruptions is currently in progress though it requires a more rooted direction. By decomposing cognitive tests into more simple and accessible constructs and using additional qualitative characteristics of the performance, one can assort the related brain activity. Since the cognitive tests performances are often characterized by multiple errors, which can also be indirectly seen from a variety of brain activations, the possible approach could be to scrutinize the performance and to match the behavioral and neural patterns with the help of the recent mathematical modeling and connectivity tools. The error monitoring system should be also taken into account when investigating the complex brain-behavioral interactions in healthy subjects and in schizophrenia patients.

## Author contributions

YZ has substantially contributed to the concept of the review, has made literature search and drafted the manuscripts. IF has contributed to the drafting of the manuscript and discussing and editing. BD, EB, IS, JM have written the parts of the manuscript. KŠ has written a part of the manuscript and edited the manuscript. MR and FŠ made critical points. JH has been involved into the conceptual discussion and provided the critical revisions of the manuscript. All authors read and approved the final manuscript.

### Conflict of interest statement

The authors declare that the research was conducted in the absence of any commercial or financial relationships that could be construed as a potential conflict of interest.

## Footnotes

^1^The Trail Making Test (TMT) is divided into two parts, in part A (TMT-A), typically the subject connects 25 encircled numbers randomly scattered on a page in ascending order by drawing a pencil line. In part B (TMT-B) there are 25 encircled numbers and letters that should be connected in alternating order.

^2^The test is administered by PC software and takes approximately 15 min. In standard CPT-X version respondent is required to respond when any letter, except the letter “X,” appears on the screen (Conners, 2014).

^3^Performance on the VFT is measured by the number of words generated within a set time limit, usually, 1 min, and normative data have been collected for different age groups, suggesting average ranges of words/min that should be expected from healthy subjects (Lezak et al., 2012).

^4^The DS test is composed of two different types, the Digit Span Forward (DSF), and Digit Span Backward (DSB), each testing different cognitive functions (Banken, 1985; Kaplan, 1991). In DSF test, the subject is requested to repeat a specific sequence of numbers, in the same order as presented. The DSB test follows the same principle and the main difference is that the subject is requested to repeat the sequence in a reverse order. DSB is an active procedure that requires effort, as it is composed by the encoding of the span, the manipulation, and reverse of the order and the recall of the correct digits, which makes it more demanding than DSF [(Banken, 1985; Black, 1986)].

^5^The standard administration of RAVLT includes five successive presentations (T1–T5) of the 15 words list (A) followed by free recall on each learning trial, (T6) presentation and recall of the “interference” list (B), a post-interference recall trial of the original list (A); and finally, the delayed recall (T7) and recognition (R) trials of the original list (A) with delay varying from 15 to 60 min (Mitrushina et al., 2005; Lezak et al., 2012; Preiss et al., 2012). Even that some studies do not apply the delayed recall or recognition trials, all parts of the test are clinically important.

^6^given over individual recall trials (T1+T2+T3+T4+T5).

^7^However, self-recognized repetitions, without an increased number of confabulations and intrusions, may be interpreted as an increased effort to recall as many words as possible (e.g., Bleecker et al., 2005).

^8^Both immediate and delayed recall and recognition in RAVLT.

^9^The study showed negative correlations between behavioral measures and the thalamus—functional connectivity of putamen and dorsomedial nucleus to thalamus and thalamus to caudate connectivity.
